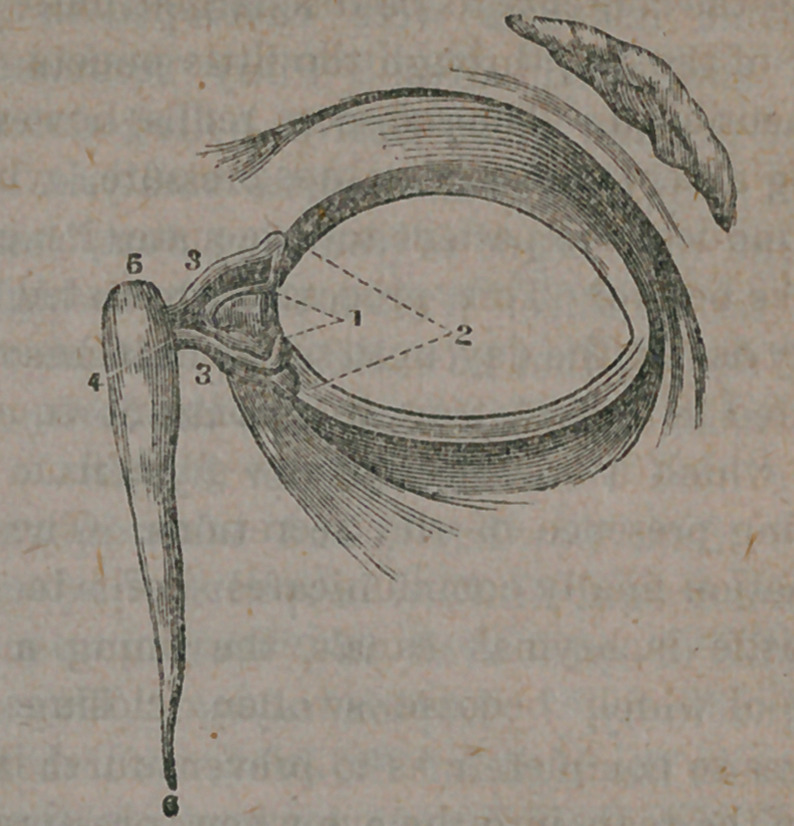# Tear Passages

**Published:** 1873-01

**Authors:** 


### TEAR-PASSAGES.

Most of our readers, perhaps, have seen persons who have been greatly annoyed by tears standing in their eyes, and flowing over the face, when exposed to cold air or winds. The occurence is quite common, and is the result of a stricture or contraction of the walls of the lachrymal or tear passages, whose office it is to carry off the tears from the eyes, after they have performed their special duty of lubricating those organs.

If you will secure the privilege of examining a friend's eye, you will discover, at the inner corner of the lids, next the nose, a very small opening, no larger than the point of a pin,—one on the under and another on the upper lid, opposite each other, at the ends of the dotted lines marked 2, in the following illustration:—

These are the puncta, or openings to the little lachrymal canals, marked 3, whose work it is to carry the tears into the lachrymal sac, which occupies the space between 4 and 5 in our figure. The tears having collected in this sac, are then discharged into the nose, through the lachrymal duct, which is that portion extending from 4 to 6, and is of about the length and size represented in our illustration. This being somewhat of a circuitous route, it is not to be wondered at that obstructions are sometimes found in the passage, from which cause the flow of the tears, through their natural course, is prevented, compelling them to find escape over the cheek, and so giving rise to several forms of disease which we are about to describe.

Inflammation of the lachrymal sac, the most frequent cause for obstruction in the duct, may arise from nasal catarrh,—an extension of the inflammation of the mucus membrane of the nose to the same lining of the sac. It is also the result of scrofulous or syphilitic taint, and will be found accompanying granular lids, and exposure to wet and cold.

People are too apt to neglect slight obstructions of the duct, supposing that no real harm is being done as long as they are willing to bear the simple annoyance of the tears flowing over the face. But this obstruction to the proper flow of the discharges, soon leads to very serious trouble and an immense amount of suffering.

The mucus membrane, lining the passage, is, at the first symptom of obstruction, merely thickened or congested, from a catarrhal attack, or perhaps, exposure while riding in an open conveyance, with face to the wind. After a few days, a little swelling is found in the corner of the eye, next the nose, slightly tender to the touch, but which can be evacuated of its tears or mucus, by slight pressure with the finger, causing the contents to be discharged into the corner of the eye, through the little puncta, before mentioned. The sac soon refills, however, causing a very uncomfortable pressure, which again induces the patient to evacuate its contents, as before. This process is repeated frequently during the day, until severe inflammation is excited in the sac, from the constant squeezing to which it is subjected, as well as from the irritating presence of the secretions. The inflammation finally communicates itself to the two little lachrymal canals, the lining membranes of which become swollen, closing the passages so completely as to prevent further escape of the tears into the eye when pressure is applied. The accumulated matter in the sac being unable to escape either through the canals or duct, excite such a high state of inflammation with consequent swelling, as to resemble erysipelas. The sac swells, pus forms, which escapes beneath the skin, producing excrutiating pain, throbbing and swelling, until both eyes are completely closed. No relief is had until the matter escapes voluntarily through the skin upon the face, or the surgeon is called in to relieve the imprisoned pus by an incision of his lancet.—When the disease is allowed to progress so far, the matter discharges itself through an opening upon the face, a permanent fistula is produced, which is not only very annoying and disgusting to observe, but is likewise very difficult to heal. Should the fistula become obstructed, which is often the case, the sac again refils and goes through another process of swelling, inflammation, pain and suppuration. By the time this formula is repeated a dozen times or more, the patient is willing to do just what should have been done in the very first instance,—consult an ophthalmic surgeon for relief.

Heretofore it has been the custom to insert leaden or silver stylets, through an opening made in the face, near the corner of the eye, through the duct into the nose. These styles are supported by means of a head, which sets upon the face, presenting rather an unsightly appearance. This process is now rarely resorted to; and when the surgeon finds it necessary to insert a style, the head is allowed to rest in an unobserved position, between the lids, at a point indicated at the end of the white line marked 4, in our figure. This is an improvement in the manner of inserting the stylet which gives better and more direct exit to the tears, at the same time removing any objection to the operation on account of its unsightliness.

But the treatment almost invariably adopted and recommended by ophthalmic surgeons at this day is, to remove any obstruction or constriction that may exist either in the canals or the duct proper, by means of a systematic and painless probing and syringing of these passages. If the patient is fortunate enough to apply for relief as soon as the constriction is made manifest, by the overflowing of tears upon the face, and the unusual "weeping" condition of the eyes, the cure is easily and painlessly accomplished by the mildest of remedies and means.—Later, it may be necessary to slit the little canals from their puncta upon the lids, to where they enter the lachrymal sac. A probe is then easily introduced into the nasal duct, and all obstruction there overcome, without the necessity for an insertion of the stylet. Should the patient defer the visit until the disease has run its course, and a fistulous opening established upon the face, considerable treatment, with the syringe, probing, and other means of healing the ulcerated sac, and dilating the passage will have to be resorted to, requiring several weeks, of daily treatment, to accomplish the cure.

We hope, therefore, our readers see the necessity for attending to the early symptoms of obstruction to the proper and free flow of the natural discharges from the eye, and so save themselves much annoyance, pain, time and money.

## Figures and Tables

**Figure f1:**